# Algorithms and Complexity Analyses for Control of Singleton Attractors in Boolean Networks

**DOI:** 10.1155/2008/521407

**Published:** 2008-06-12

**Authors:** Morihiro Hayashida, Takeyuki Tamura, Tatsuya Akutsu, Shu-Qin Zhang, Wai-Ki Ching

**Affiliations:** 1 Bioinformatics Center, Laboratory of Biological Information Networks, Bioinformatics Center, Institute for Chemical Research, Kyoto University, Uji, Kyoto 611-0011, Japan; 2 School of Mathematical Sciences, Fudan University, Shanghai 200433, China; 3 Advanced Modeling and Applied Computing Laboratory, Department of Mathematics, The University of Hong Kong, Pokfulam Road, Hong Kong

## Abstract

A Boolean network (BN) is a mathematical model of genetic networks. We propose several algorithms for control of singleton attractors in BN. We theoretically estimate the average-case time complexities of the proposed algorithms, and confirm them by computer experiments. The results suggest the importance of gene ordering. Especially, setting internal nodes ahead yields shorter computational time than setting external nodes ahead in various types of algorithms. We also present a heuristic algorithm which does not look for the optimal solution but for the solution whose computational time is shorter than that of the exact algorithms.

## 1. Introduction

One of the important challenges of computational systems biology and bioinformatics is to develop a control theory for biological systems [1,2]. Development of such a control theory is interesting from both a theoretical viewpoint and a practical viewpoint. From a theoretical viewpoint, biological systems are highly nonlinear. For control of linear systems, extensive studies have been done, and rigorous theories and useful methods have been developed. Furthermore, many of these methods have been applied to control various kinds of real systems. However, it is recognized that control of nonlinear systems is far more difficult than control of linear systems. Though there are some established methods for control of nonlinear systems [3,4], these can only be applied to certain classes/special cases. In particular, it is very difficult to control large-scale nonlinear systems. From a practical viewpoint, as Kitano wrote [1,2], identification of a set of perturbations that induces desired changes in cellular behaviors may be useful for systems-based drug discovery and cancer treatment. For example, Takahashi (this author along with Morihiro Hayashida contributed equally to this work) and Yamanaka developed induced pluripotent stem cells (iPS cells) by introducing 4 kinds of transcription factors (Oct3/4, Sox2, c-Myc, Klf4) into fibroblast cells of mouse [5]. Furthermore, Takahashi et al. [6] and Yu et al. [7] independently succeeded to develop iPS cells by introducing 4 kinds of factors into human cells. It is to be noted that Yamanaka et al. introduced 4 transcription factors of Oct3/4, Sox2, c-Myc, and Klf4 into fibroblast cells, whereas Thomson et al. introduced 4 factors of OCT4, SOX2, NANOG, and LIN28 into somatic cells. Though these seminal discoveries were achieved based on their knowledge, experience, and many experiments, systematic methods might help such kind of works. Therefore, we study systematic methods for control of biological systems. In this paper, we focus on control of gene regulatory networks because these networks play a fundamental role in cells and may be efficiently controlled by overexpression and suppression of genes.

Various kinds of mathematical models have been proposed for modeling gene regulatory networks. These models include neural networks, differential equations, Petri nets, Boolean networks, probabilistic Boolean networks (PBNs), and multivariate Markov chain model [8–11]. Among these models, *Boolean network* (BN) [12–14] has been well studied. BN is a very simple model; each node (e.g., gene) takes either 0 (inactive) or 1 (active), and the states of nodes change synchronously. Although BN is very simple, its dynamic process is complex and can give insight into the global behavior of large genetic regulatory networks [15].

The total number of possible global states for a Boolean network with  genes is . However, for any initial condition, the system will eventually evolve into a limited set of stable states called *attractors*. The set of states that can lead the system to a specific attractor is called the *basin of attraction*. Each attractor can contain one or many states. An attractor having only one state is called a *singleton attractor*. Otherwise, it is called a *cyclic attractor*. Attractors are biologically interpreted so that different attractors correspond to different cell types [14] or different cell states [16].

Motivated by this biological interpretation, extensive studies have been done on the average-case analysis of the number and length of attractors in randomly generated BNs [14,17–19], although there is no conclusive result. Recently, several methods have been developed for efficiently finding or enumerating attractors in BNs [20–23], whereas it is known that finding a singleton attractor (i.e., a fixed point) is NP-hard [24,25]. Devloo et al. developed a method using transformation to a constraint satisfaction problem [20]. Garg et al. developed a method based on binary decision diagrams (BDDs) [21]. Irons developed a method that makes use of small subnetworks [22]. However, theoretical analysis of the average-case complexity was not addressed in these works. We recently developed algorithms for identifying singleton attractors and small attractors, and analyzed the average-case time complexities of these algorithms [23].

Finding a sequence of control actions for BNs is another important topic on BNs. Datta et al. proposed methods for finding control actions for probabilistic Boolean networks (PBNs) [26–28], where a PBN is a probabilistic extension of a BN [29]. In their approach, the control problem is defined as minimization of the total of control cost and the cost of terminal state. The control cost is defined as the cost of applying control inputs in some particular states, and higher terminal costs are usually assigned to those undesirable states. Their approach is based on the theory of controlled Markov chains, and makes use of the theory of probabilistic dynamic programming. They extended their approach for handling context-sensitive PBNs [30] and/or infinite-horizon optimal control [31]. Since BNs are special cases of PBNs, their methods can also be applied to finding control actions for BNs. However, all of these approaches need to handle  matrices, which limits application of these approaches only to small size (e.g., less than 20 nodes) networks. Therefore, we studied computational complexity of the control problem on BN and PBN, and proved that finding an optimal control strategy is NP-hard for both BN and PBN [32]. In order to break the barrier of computational complexity, an approximate finite-horizon optimal control has been introduced [33] and a heuristic method based on -learning algorithm for approximating the optimal infinite-horizon control policy has been proposed [34]. However, application of these approaches is still limited to small networks.

In this paper, we propose a new model for control of BN, that is, *control of attractors* of BN. Though our model can be extended to cyclic attractors to some extent (as shown in Section 3.9), here we focus on singleton attractors. Since cyclic attractors correspond to cell cycles appearing in such cases as cell division and cell growth whereas singleton attractors correspond to steady states of cells or cell types, it is reasonable to begin with singleton attractors. We assume that a BN and a score function are given as an input, where the score function indicates the closeness of the attractor state to the desired state. We also assume that nodes in a BN are divided into *internal nodes* and *external nodes*, where states of external nodes can only be controlled. Then, our objective is to determine 0/1 states of external nodes so that the score of the resulting singleton attractor is maximized. However, if there exist multiple attractors, the attractor into which a BN is evolved depends on an initial state of a BN. Since it is very difficult to know the initial state exactly, we modify the objective so that the minimum score of the singleton attractors is maximized or exceeds a given threshold. In this model, external nodes correspond to candidate genes and/or transcription factors to be added or to be deleted (suppressed), and the objective is to make a cell to go to a preferable state regardless of the current state of the cell.

In order to solve the proposed problem, we develop several algorithms based on our previous work [23]. In [23], we developed a series of algorithms for finding singleton and small attractors in a BN. The most important feature of the algorithms is that the average-case time complexity was theoretically analyzed and was experimentally corroborated. It was shown that most of these are much faster than  if the maximum indegree is bounded by some constant . For example, one of the algorithms works in  time and  time (in the average case) for  and  respectively, which are much faster than . Many of the algorithms proposed in this paper have similar properties. For example, it is shown that one of the algorithms works in  and  times for  and  respectively, under some reasonable conditions. Though these time complexities are worse than those in [23], the problem considered in this paper is much more difficult than the one in [23]. Therefore, these results are reasonable and are still much faster than . It is to be noted that some of the proposed algorithms are far from straightforward extensions of [23], and novel ideas are introduced in some of the theoretical analyses. Most of the theoretical results are corroborated through computational experiments.

It is to be noted that the state-space-based methods [26–28,31,33] need at least  time. Though a -learning-based method [34] needs polynomial update time, it seems that an exponential number of repetitions are required to obtain preferable control actions. Our proposed model may be interpreted as a variant of the infinite-horizon control model [31]. However, our developed algorithms are quite different from those in [31]. Though our proposed algorithms are based on [23], the problems to be solved are different from those in [23] and several new ideas are introduced in development of the algorithms. As a related work, Pal et al. studied the problem of generating BNs with a prescribed attractor structure [28]. Though their model has some similarity with our model, applicability of their methods is limited to small size networks.

The organization of the paper is as follows. First, we briefly review BN and then give a formal definition of the problem. Next, we present our proposed algorithms, their theoretical analyses, and the results on computational experiments. Then, we present an approximate but faster heuristic algorithm. Finally, we conclude with future work.

## 2. Problem of Controlling Singleton Attractors

In this section, we briefly review the Boolean network model, and then formulate the problem explained above. After that we present enumeration-based algorithms and perform theoretical and empirical analyses.

### 2.1. Boolean Network and Attractor

Let  represent a Boolean network which consists of a set of  nodes, , and  Boolean functions, . Generally,  and  are regarded as genes and a set of regulatory rules of genes, respectively. Let  denote the state of  at the time step , where  means that the th gene is not expressed, and  means that it is expressed. The overall expression level of all genes in the BN is represented by , which is called the *gene activity profile* (GAP) of the network at time . Since  ranges from  to , there are  possible global states. Regulatory rules of gene states are given as follows:(1)

This rule means that the state of gene  at time  depends on the states of  genes at time , where  is called the *indegree* of . Furthermore, the maximum indegree of a BN is defined as . The number of genes which are directly influenced by gene  is called the *outdegree* of gene . The states of all genes are changed synchronously according to the corresponding Boolean functions. A consecutive sequence of GAPs () is called an attractor with period  if . When , an attractor is called a *singleton attractor*. When , it is called a *cyclic attractor*.

**Figure 1:**
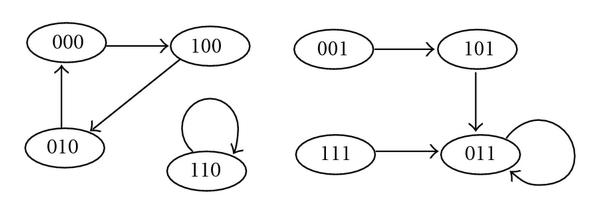
**State transitions of the Boolean network shown in Table 1**.

An example of a truth table of a BN is shown in Table 1. Every gene  updates its state according to a regulatory rule . Since the state transitions of this BN are as shown in Figure 1, the system will eventually evolve into one of three attractors. Two of them are singleton attractors,  and . The other is a cyclic attractor with period 3, .

**Table 1:** Example of a truth table of a Boolean network.

					
0	0	0	1	0	0
0	0	1	1	0	1
0	1	0	0	0	0
0	1	1	0	1	1
1	0	0	0	1	0
1	0	1	0	1	1
1	1	0	1	1	0
1	1	1	0	1	1

In this paper, we assume that there are two types of nodes in a BN: *external nodes* and *internal nodes*. Let  and  be external and internal nodes of a BN, respectively. Note that the total number of nodes in a BN is  hereafter. When it is not necessary to distinguish internal and external nodes,  are used to specify nodes. Furthermore, let  and  denote  and , respectively.

Now, we formulate the main problem of this paper.

### 2.2. Singleton Attractor Controlling Problem (SACP)

(i) *Input*: a Boolean network which consists of  external nodes and  internal nodes, and a score function , that is, a function from  to real. We assume that Boolean functions are randomly assigned to nodes and that the parent nodes of each node are also randomly determined with .

(ii) *Output*: a 0-1 assignment to external nodes, which maximizes the minimum score of singleton attractors, where the score of an attractor is given as .

**Figure 2:**
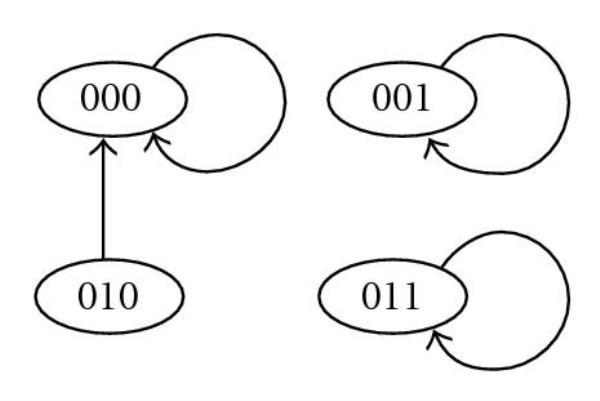
**State transitions of the Boolean network shown in Table 3**.

**Figure 3:**
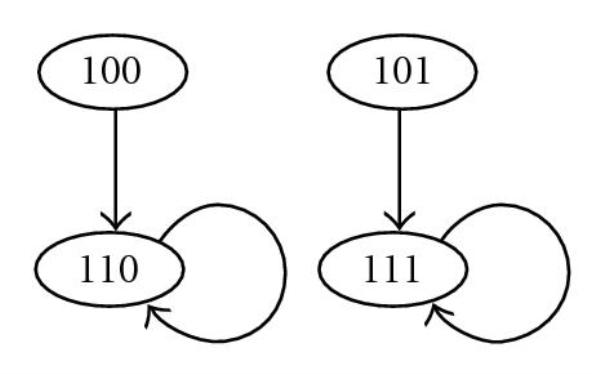
**State transitions of the Boolean network shown in Table 4**.

For example, in a BN of Table 1, let  be an external node and let  and  be internal nodes. Furthermore, assume that score functions of nodes of this BN are given as in Table 2. If  is fixed as 0, the BN of Table 1 is converted to that shown in Table 3, and its state transition is shown in Figure 2. In this BN, there are three singleton attractors, , , and , and their scores are , , and , respectively. Therefore, when  is fixed as 0 in the BN of Table 1, the minimum score of singleton attractors is 6. On the other hand, if  is fixed as 1, the BN of Table 1 is converted to that shown in Table 4, and its state transition is shown in Figure 3. In this BN, there are two singleton attractors,  and , and their scores are  and , respectively. Therefore, when  is fixed as 1 in the BN of Table 1, the minimum score of singleton attractors is 7. Thus, in order to maximize the minimum score of singleton attractors, we should fix the external node  as 1 since .

**Table 2:** Example of a score function of a Boolean network.

			
0	3	1	2
1	0	5	4

**Table 3:** If  is fixed as 0 in the truth table of Table 1, the following one is obtained.

					
0	0	0	0	0	0
0	0	1	0	0	1
0	1	0	0	0	0
0	1	1	0	1	1

**Table 4:** If  is fixed as 1 in the truth table of Table 1, the following one is obtained.

					
1	0	0	1	1	0
1	0	1	1	1	1
1	1	0	1	1	0
1	1	1	1	1	1

For this problem, one of the robust algorithms is to enumerate all singleton attractors and check the score of every singleton attractor. For this strategy, it is reasonable to utilize the basic recursive algorithm [23] as a subroutine. Although algorithms proposed in this paper are to some extent similar to those in [23], further observations and different approaches are necessary to estimate their computational time since [23] does not include the notion of external and internal nodes.

## 3. Enumeration-Based Algorithms

Before presenting enumeration-based algorithms for SACP, we briefly review the basic recursive algorithm in [23]. In this algorithm, partial GAPs are extended one by one towards a complete GAP according to a given gene ordering. If it is found that a partial GAP cannot be extended to a singleton attractor, the next partial GAP is examined. Although all proposed algorithms in this section are based on the same framework which includes the basic recursive algorithm as a subroutine, gene orderings are different from each other. Therefore, we explain only methods of gene ordering for most algorithms although we present the whole pseudocode of the first algorithm.

In what follows, we present algorithms for SACP and estimate their average computational time. Since some approximations are used for these theoretical analyses, each estimated computational time is not exactly the same as the result of the computer experiments shown in Section 3.8.

### 3.1. Algorithm 1: ExternalAhead

Theoretical Analysis

Assume that  of  internal nodes have already been examined. The overall computational time can be represented by(2)

The number of terms is , and each term will be exponential function of  as shown below. The overall average time complexity will only be affected by the largest term in (2) since  holds for arbitrary  when  and  is large enough. Similar discussions will also be applied to the other algorithms.

For internal nodes, we have(3)

The probability that the algorithm examines the th gene is not more than(4)

The number of recursive calls executed for the first  genes is at most(5)

By setting , we can obtain . Furthermore, we assume that . Therefore, (5) is rewritten as(6)

Thus, the average computational time can be estimated as(7)

With simple numerical calculations, we can confirm that the maximum values of (6) for fixed  and  are as shown in Tables 5 and 7.

**Table 5:** Theoretical time complexities for .

	ExAhead	Basic	ExBehind	ExLastOne	LastOneAny	LastOne
0.01						
0.02						
0.03						
0.04						
0.05						
0.06						
0.07						
0.08						
0.09						
0.10						
0.167						
0.20						
0.30						
0.333						

### 3.2. Algorithm 2: Basic

*Algorithm for gene ordering.* Nodes are chosen at random.

Theoretical Analysis

Assume that  of  external nodes and  of  internal nodes have already been examined. We can assume that  holds approximately. When  is large (compared with ),(8)

The probability that the algorithm examines the th gene is not more than(9)

The number of recursive calls executed for the first  genes is at most(10)

Note that the above term can be ignored when  is small. By setting  and , the above term can be rewritten as(11)

By setting ,(12)

Similar to the analysis of the previous algorithm, the average computational time can be estimated as  and its maximum values for fixed  and  are shown in Tables 5 and 7. Note that the range of  is different from that of the previous algorithm.

Intuitively, this algorithm is the same as the basic recursive algorithm in [23]. However, the computational time depends on  since  always holds for an external node. Therefore, assigning an external node always leads to the next recursive loop, and thus the computational time becomes higher than that of the basic recursive algorithm in [23].

### 3.3. Algorithm 3: ExternalBehind

*Algorithm for gene ordering*. First all internal nodes are examined (Step 1). After that all external nodes are examined (Step 2).

Theoretical Analysis

At Step 1, the number of recursive calls executed for the first  genes is at most(13)

By setting , we can obtain . Note that the definition of  is different from those of the previous algorithms. Therefore,(14)

Furthermore, by setting ,(15)

At Step 2, the number of recursive calls executed for the first  genes is at most(16)

By setting ,(17)

The whole computational time of ExternalBehind can be bounded by(18)

It can be confirmed that the maximum values for fixed  and  are as shown in Tables 5 and 7.

### 3.4. Algorithm 4: ExternalLastOne

To achieve smaller time complexity, it is necessary to detect a contradiction for the condition of a singleton attractor at early stage. To detect a contradiction from a node, the node and all its parent nodes must be assigned. Therefore, one of the reasonable methods is to find an assigned node  for which  of  parent nodes have already been assigned, and then assign the nonassigned node so that all parent nodes of  are assigned. We call such a nonassigned node *LastOne* node. In the following three algorithms, we utilize the notion of "LastOne." The frameworks of these three algorithms are the same. (i) First, a nonassigned node is randomly chosen. (ii) Second, if there is a "LastOne" node, assign it either 0 or 1. By further restricting (i) and (ii), we developed the following three algorithms as shown in Table 9.

*Algorithm for gene ordering*. If there is an external node  which satisfies the following condition,  is chosen to be assigned either 0 or 1. Otherwise, a nonassigned internal node is randomly chosen. *and all parent nodes of**have already been assigned except*.

If there are multiple external nodes and both of them satisfy the condition, one of them is randomly selected to be assigned. Moreover, if some external nodes are still nonassigned when all internal nodes have been assigned, remaining nodes will be randomly chosen one by one.

Example 3.1.

**Figure 4:**
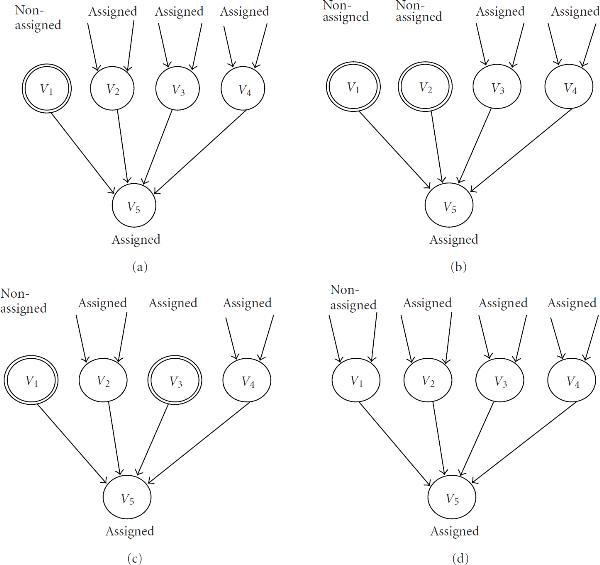
**Example for gene ordering**.

Assume that , , , and  have already been assigned either 0 or 1 as shown in Figure 4(a). Furthermore, assume that  is an external node and has not been assigned yet. In such a case, we select  instead of randomly selecting a nonassigned internal node.

For another example, assume that , , and  have been assigned as shown in Figure 4(b). Moreover, assume that both  and  are nonassigned external nodes. If all internal nodes have already been assigned at this point, one of  and  will randomly be chosen to be assigned and then the other will be assigned. However, such a case rarely happens since  is small.

Theoretical Analysis

Assume that  of  external nodes and  of  internal nodes have already been assigned. The average number of edges which are from internal nodes to  is . The average number of internal nodes of which all parent internal nodes have already been assigned is(19)

Since the average outdegree of an external node is also ,(20)

holds approximately. Therefore, we have(21)

By setting  and ,(22)

holds.

On the other hand,(23)

holds when  is small. Therefore, the probability that ExternalLastOne examines the next internal node of  is not more than(24)

The number of recursive calls executed for the first  nodes is at most(25)

by setting  and . From (22) and (25), the computational time of ExternalLastOne can be bounded by(26)

It can be confirmed that the maximum values for fixed  and  are as shown in Tables 5 and 7.

### 3.5. Algorithm 5: LastOneAny

*Algorithm for gene ordering*. If there is a node  of which all parent nodes have already been assigned except ,  will be selected to be assigned either 0 or 1. Otherwise, a nonassigned node is randomly chosen to be assigned. If there are multiple nodes and both of which satisfy the above condition, one of them is randomly selected to be assigned.

Example 3.2.

Assume that , , , and  have already been assigned either 0 or 1 as shown in Figure 4(c). Furthermore, assume that  has not been assigned yet. In such a case, we select  instead of randomly selecting a nonassigned node. Note that  is not limited to an external node. Moreover, external nodes and internal nodes are not distinguished in this algorithm at all.

Theoretical Analysis

We have that(27)

holds when  is small. The probability that LastOneAny examines the th gene is not more than(28)

The number of recursive calls executed at this step is at most(29)

by setting , , and . Thus, the average computational time can be estimated as(30)

With simple numerical calculations, we can confirm that the maximum values of (30) for fixed  and  are as shown in Tables 5 and 7.

### 3.6. LastOne

*Algorithm for gene ordering*. If there is a node  which satisfies the following condition,  is chosen to be assigned either 0 or 1. Otherwise, a nonassigned internal node is randomly chosen. *and all its parent nodes have been assigned except*.

If there are multiple nodes and both of which satisfy the above condition, one of them is randomly selected to be assigned.

Example 3.3.

Assume that , , , and  have already been assigned either 0 or 1 as shown in Figure 4(d). Furthermore, assume that  has not been assigned yet. In such a case, we select  instead of randomly selecting a nonassigned internal node. Note that  is not limited to an external node, but external nodes and internal nodes are distinguished when nonassigned nodes are randomly selected.

Theoretical Analysis

Since the average outdegree of an external node is also ,(31)

holds approximately. Therefore, we have(32)

By setting  and ,(33)

holds.

On the other hand, the probability that LastOne examines the th gene is not more than(34)

The number of recursive calls executed for the first  genes is at most(35)

by using . From (33) and (35), the average computational time can be estimated as(36)

With simple numerical calculations, we can confirm that the maximum values of (36) for fixed  and  are as shown in Tables 5 and 7.

### 3.7. OutdLastOne

In addition to the above algorithms, we tried to find faster algorithms for SACP in terms of empirical time complexity. As a result, the following algorithm yielded the best as shown in Tables 6 and 8, although theoretical analysis has not been performed. This algorithm is the extension of "outdegree-based algorithm" of [23].

**Table 6:** Empirical time complexities for .

	ExAhead	Basic	ExBehind	ExLastOne	LastOneAny	LastOne	OutdLastOne
0.10							
0.167							
0.20							

**Table 7:** Theoretical time complexities for .

	ExternalAhead	Basic	ExternalBehind	ExternalLastOne	LastOneAny	LastOne
0.01						
0.02						
0.03						
0.04						
0.05						
0.06						
0.07						
0.08						
0.09						
0.10						
0.167						
0.20						
0.30						
0.333						

**Table 8:** Empirical time complexities for .

	ExAhead	Basic	ExBehind	ExLastOne	LastOneAny	LastOne	OutdLastOne
0.10							
0.167							
0.20							

**Table 9:** ExternalLastOne, LastOneAny, and LastOne.

	(ii) is applied to only external nodes	(ii) is applied to both external and internal nodes
(i) is applied to only internal nodes	ExternalLastOne	LastOne
(i) is applied to both external and internal nodes		LastOneAny

*Algorithm for gene ordering*. If there is a node  which satisfies the following condition,  is chosen to be assigned either 0 or 1. Otherwise, a nonassigned internal node with the highest outdegree is randomly chosen. *and all its parent nodes have been assigned except*.

If there are multiple nodes and both of which satisfy the above condition, the one with the highest outdegree is randomly selected to be assigned.

Example 3.4.

Assume that , , , and  have already been assigned either 0 or 1 as shown in Figure 4(d). Furthermore, assume that  has not been assigned yet. In such a case, we select  instead of randomly selecting an internal node with the highest outdegree.

### 3.8. Computer Experiments for Enumeration-Based Algorithms

**Figure 5:**
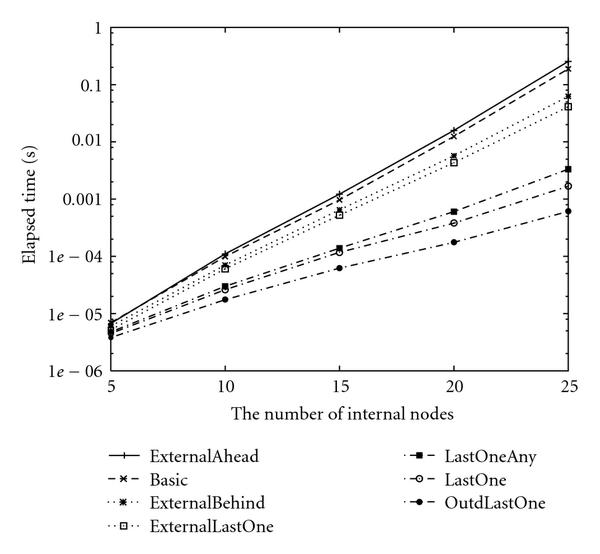
**Elapsed time of enumeration-based algorithms for SACP with  and **.

In this section, we evaluate the proposed algorithms by performing computer experiments on random networks, and compare empirical time complexities with theoretical ones. We randomly generated 100 Boolean networks with indegree , and took the average values. These computational experiments were done on a PC with Xeon 3.6 GHz CPUs and 3 GB RAM under the Linux (version 2.6.16) operating system, where the icc compiler (version 10.1) was used with optimization option-O3-ipo. For each  and each , we plotted 4 or 5 points for each method. For example, Figure 5 shows the experimental result for , . In the experiment, we randomly generated 100 Boolean networks for . We used a tool for GNUPLOT to fit the function  to the logarithms of the experimental results. The tool uses the nonlinear least-squares (NLLSs) Marquardt-Levenberg algorithm.

**Figure 6:**
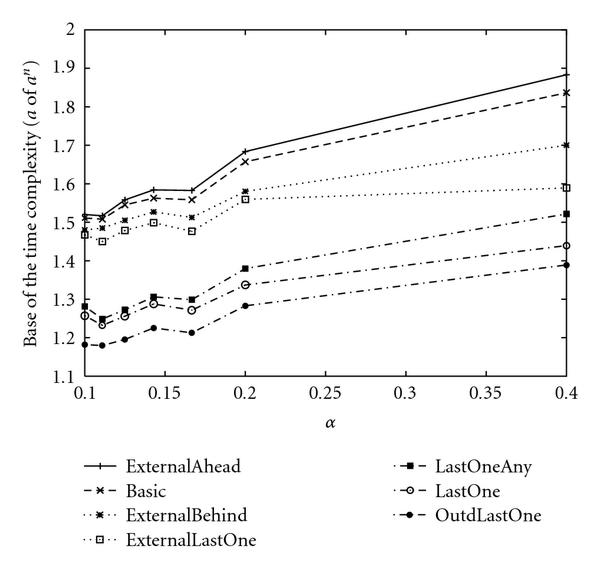
**Base of the empirical time complexities of the enumeration-based algorithms for SACP with **.

**Figure 7:**
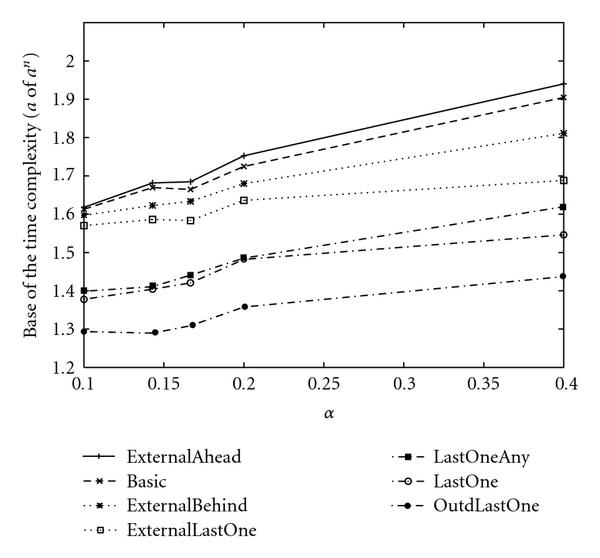
**Base of the empirical time complexities of the enumeration-based algorithms for SACP with **.

As a result, empirical time complexities for each algorithm with  and  are shown in Tables 6 and 8. Since some approximations are used in the theoretical analyses, the theoretical time complexities shown in Tables 5 and 7 are not exactly the same as those of empirical time complexities shown in Tables 6 and 8. However, magnitude correlations of these algorithms are the same for each  and . Furthermore, differences between theoretical time complexities and empirical time complexities are not very large for each  and . Thus, we can say that our estimation of the theoretical time complexity of each algorithm is relatively appropriate although we used several theoretical approximations to estimate them.

### 3.9. Comparison among Proposed Algorithms

As a result of theoretical and empirical analyses for the proposed algorithms for SACP, if  is not large, it is seen that "LastOne  LastOneAny  ExternalLastOne  ExternalBehind  Basic  ExternalAhead" holds in terms of necessary computational time, where A  B means that A is faster than B. One of the reasonable methods for analyzing the above result is to distinguish these algorithms by depending on whether external nodes or internal nodes are assigned first.

Let us classify these algorithms into the following three types. (i) First, assign internal nodes. After that assign external nodes. (ii) First, assign external nodes. After that assign internal nodes. (iii) Do not distinguish internal and external nodes. From " ExternalBehind  Basic  ExternalAhead", it is seen that (i)  (iii)  (ii) holds for the most basic type of algorithms. Although the other algorithms utilize the notion of "last one," they can also roughly be classified into the above three types. For example, the only difference between "LastOne" and "LastOneAny" is that "LastOne" randomly selects only internal nodes when there are no special nodes, whereas "LastOneAny" randomly selects nodes from both internal and external nodes in the same condition. Therefore, it is reasonable to regard "LastOne" and "LastOneAny" as (i) and (iii), respectively, when comparing these two and we can confirm that (i)  (iii) holds again. On the other hand, the only difference between "ExternalLastOne" and "LastOne" is that the notion of "last one node" is only applied to external nodes in "ExternalLast," whereas the notion is applied to both internal and external nodes in "LastOne". Therefore, it is also reasonable to regard "ExternalLastOne" and "LastOne" as (ii) and (iii), respectively, in this comparison, and we can confirm that (iii)  (ii) holds. Note that "LastOne" is classified into (i) in the previous comparison but is classified into (iii) this time. It depends on which two are compared. Thus, we can confirm that (i)  (iii)  (ii) holds for various types of comparisons. Intuitively, to reduce the computational time, it is necessary to detect a contradiction for the condition of a singleton attractor at early stage. To detect a contradiction from a node, the node and all its parent nodes must be assigned. However, since  always holds for an external node, algorithms cannot detect the contradiction from external nodes. That is why assigning internal nodes first reduces the computational time.

However, if cyclic attractors are taken into consideration, the above property does not hold. Now, we formulate the extended version of SACP as follows.

ACP: Attractor Controlling Problem

(i) *Input*: a Boolean network which consists of  external nodes and  internal nodes, and a score function , that is, a function from  to real. We assume that Boolean functions are randomly assigned to nodes, and parent nodes of each node are also randomly determined with .

(ii) *Output*: a 0-1 assignment to external nodes, which maximizes the minimum score of attractors whose periods are , where . The score of an attractor is given as .

Note that the score of a cyclic attractor is defined as the sum of the score of GAP for each , but it can be extended to other definitions such as the sum of the minimum score of each node.

Although our proposed algorithms were introduced for SACP, we extended and implemented them for ACP(2) and ACP(3). A pseudocode of ExternalAhead for ACP is shown in Algorithm 2. Although the main part of each algorithm is the same as that for SACP, the process for checking whether the partial assignments contradict the condition of attractors is different. Let *x-ancestor* of  be nodes which have a directed path to  with length less than or equal to . For SACP, algorithms only check the relationship between the assignment of each node and its parent nodes. However, for ACP, algorithms check the relationship between the assignment of each node and its -ancestors.

Empirical time complexities for ACP(2) and ACP(3) are shown in Tables 10 and 11, respectively. Since the number of - is relatively large when compared with  (around 30) for ACP(3), some elements in Table 11 are larger than . Note that these values would be less than  if  were much larger. It seems that (ii)  (iii)  (i) holds for  since "ExternalAhead  Basic  ExternalBehind" holds in Tables 10 and 11 although the complexities of "LastOne" and "LastOneAny" are almost the same. It seems that the number of -ancestors affects the empirical time complexities largely. For example, "ExternalAhead" is the slowest for SACP but faster than "Basic" and "ExternalBehind" for ACP(2) and ACP(3). We believe that the reason is that the number of -ancestors of assigned nodes for "ExternalAhead" is smaller than that for "Basic" and "ExternalBehind" in the cases of ACP(2) and ACP(3), but it is larger in the case of SACP.

**Table 10:** Empirical time complexities for ACP(2) with .

	ExAhead	Basic	ExBehind	ExLastOne	LastOneAny	LastOne	OutdLastOne
0.10							
0.167							
0.20							

**Table 11:** Empirical time complexities for ACP(3) with .

	ExAhead	Basic	ExBehind	ExLastOne	LastOneAny	LastOne	OutdLastOne
0.10							
0.167							
0.20							

**Algorithm 1:***Algorithm for gene ordering*. First, all external nodes are examined. After that all internal nodes are examined.

Pseudocode

* ***Input**: Boolean network  and score function 

* ***Output**: 0-1 assignment to external nodes, which maximizes the minimum score of singleton attractors.

Begin

* ***Initialize**.

* ***For****to****do**

*  ***For****to****do**

*   * the th digit of the binary number representation of .

*  ***Initialize**; .

*  ***Procedure**

*   ***If** and , **then**

*    *;

*   ***for****to****do**;

***    * if** it is found that  for some , **then continue**;

*    ***else**;

*  ***if** and ,

*   ***then**;

*   ***for****to****do**

*    *;

* ***if**, **then return**;

*** * else return** null.

End

**Algorithm 2:** Pseudocode of ExternalAhead for ACP.

* ***Input:** a Boolean network  and score functions 

* ***Output:** 0-1 assignments to external nodes, which maximize the minimum score

* *of attractors whose periods are , where . The score of an attractor is given as

* *.

Begin

* ***Define**: nodes which have length- paths to .

* ***Initialize**;

* ***for****to****do**

*  ***for****to****do**

*   * the th digit of the binary number representation of .

*  ***Initialize**; .

*  ***Procedure**

*   ***If** and , **then**

*    *;

*   ***for****to****do**

*    *

*    *

*    ***for** to **do**

*     *

*     ***while** and **do**

*      ***if** every  is assigned and **then**

*       *

*      *

*    ***if****then continue**;

*    ***else**;

*  ***if** and ,

*   ***then**;

*   ***for****to****do**

*    *

* ***if**, **then return**;

* ***else return** null.

End

### 3.10. SACP in Scale-Free BN

It is known that gene regulatory networks have the scale-free property; that is, the degree distribution approximately follows the power law [35]. Moreover, it is observed that the outdegree distribution follows the power law and the indegree distribution follows the Poisson distribution [36]. We implemented OutdLastOne for SACP with scale-free networks, where indegrees are 2 and outdegrees are proportional to . (Note that this  does not mean indegrees.) The average empirical time complexities of randomly generated 100 BNs are shown in Table 13, and we can confirm that OutdLastOne in scale-free networks is almost as fast as OutdLastOne in random networks examined in Section 3.8.  were used for , and similar numbers of nodes were also used for .

## 4. Heuristic Algorithms for SACP

In the previous section, we analyzed enumeration-based algorithms for SACP. Although these algorithms are guaranteed to output optimal solutions, it may not be necessary to find the rigorous optimal solutions in some practical cases. One of the possible approaches for this purpose is to use a threshold. Based on it, we develop heuristic algorithms by modifying the original algorithms. In the original algorithms, we update the minimum score whenever a new singleton attractor is found. Instead, in the modified algorithms, we compare the score of a new singleton attractor with a given threshold  and output the corresponding assignment to external nodes as an approximate solution if the score is greater than . Of course, there may exist multiple attractors for each assignment to external nodes, and the minimum is taken (per assignment to external nodes) in the original algorithms. However, it is known that the expected number of singleton attractors is 1 [37,38]. Thus, it is expected that we can obtain a good solution even if we stop the algorithms as soon as a singleton attractor whose score is greater than  is found. How to select  is also an important issue in these heuristic algorithms. If we know appropriate  in advance, we can simply use such . Otherwise, we may examine several values of  from lower to upper. For each , we manually inspect the solution and we stop further examinations if the solution is satisfactory.

Since there is no performance guarantee on the proposed heuristic approach, we examined it by means of computational experiments. We implemented one of the proposed heuristic algorithms assuming that  is distributed in  uniformly. Furthermore, let us call the following property *selectivity*: When  is to be assigned, if  holds,  is examined in advance of examining . On the other hand, if  holds,  is examined in advance of examining . Note that the results in Tables 6 and 8 were not with selectivity.

**Figure 8:**
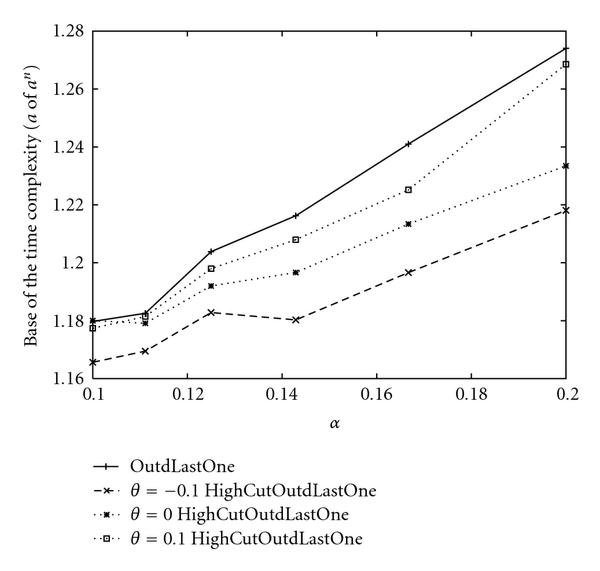
**Base of the empirical time complexities of OutdLastOne for SACP with  and **.

Since OutdLastOne was the fastest among our proposed algorithms for SACP, we implemented OutdLastOne with selectivity and , where  means that a threshold is not used. As a result, empirical time complexities for each  and  are obtained as shown in Figure 8 and Table 12, and we can confirm that using a smaller threshold yields better time complexities than using a bigger threshold or not using a threshold. Furthermore, from Tables 14 and 15, it is seen that the average number of singleton attractors in a BN is less than 1 with . Therefore, it is reasonable that the proposed algorithm stops as soon as it finds a singleton attractor whose score is greater than . Tables 14 and 15 also show the average and standard deviations of  for each case. It is seen that  is very close to  when . On the other hand,  is much smaller than  when . However, it often occurs that the algorithm cannot find desired singleton attractors when . For example, from Table 14, when , , and , it is seen that the algorithm can always find desired singleton attractors if they exist. On the other hand, when the algorithm is applied to 100 random BNs with , , , it can find desired singleton attractors only for 14 BNs although 64 of 100 BNs include singleton attractors.

**Table 12:** Empirical time complexities of OutdLastOne for  with  and selectivity.

	Without			
0.1				
0.111				
0.125				
0.143				
0.167				
0.2				

**Table 13:** Empirical time complexities of OutdLastOne for SACP in scale-free network.

	OutdLastOne
0.1	
0.167	
0.2	

**Table 14:** Average and standard deviations of  by OutdLastOne for SACP with  and selectivity.

		Average of	Standard deviation of	The number of all singleton attractors in 100 BNs	The number of singleton attractors whose scores are more than in 100 BNs
0.1	−0.1	0.0228	0.0245	64	64
(	0.0	0.0176	0.0242	64	54
)	0.1	0.0046	0.0124	64	14
0.111	−0.1	0.0271	0.0216	66	66
(	0.0	0.0235	0.0217	66	58
)	0.1	0.0060	0.0133	66	18

0.125	−0.1	0.0329	0.0323	66	66
(	0.0	0.0218	0.0281	66	51
)	0.1	0.0060	0.0195	66	12

0.143	−0.1	0.0260	0.0252	70	70
(	0.0	0.0213	0.0235	70	63
)	0.1	0.0064	0.0164	70	23

0.167	−0.1	0.0294	0.0278	73	73
(	0.0	0.0252	0.0272	73	66
)	0.1	0.0050	0.0118	73	27

0.2	−0.1	0.0340	0.0347	66	66
(	0.0	0.0305	0.0325	66	61
)	0.1	0.0146	0.0236	66	37

**Table 15:** Average and standard deviations of  by OutdLastOne for SACP with  and selectivity.

		Average of	Standard deviation of	The number of all singleton attractors in 100 BNs	The number of singleton attractors whose scores are more than in 100 BNs
0.1	−0.1	0.0552	0.0407	94	94
(	0.0	0.0443	0.0388	94	85
)	0.1	0.0108	0.0300	94	21
0.111	−0.1	0.0582	0.0403	95	95
(	0.0	0.0476	0.0395	95	91
)	0.1	0.0147	0.0329	95	31

0.125	−0.1	0.0619	0.0466	93	93
(	0.0	0.0462	0.0422	93	86
)	0.1	0.0199	0.0354	93	41

0.143	−0.1	0.0680	0.0405	94	94
(	0.0	0.0526	0.0450	94	91
)	0.1	0.0212	0.0376	94	32

0.167	−0.1	0.0619	0.0448	94	94
(	0.0	0.0528	0.0441	94	89
)	0.1	0.0206	0.0344	94	44

0.2	−0.1	0.0782	0.0502	96	96
(	0.0	0.0661	0.0474	96	92
)	0.1	0.0300	0.0450	96	58

We also implemented ExternalAhead with selectivity and a threshold for SACP. As shown in Table 16, empirical time complexities for ExternalAhead were much larger than those of OutdLastOne with  and . It is seen that assigning internal nodes first and utilizing the notion of "LastOne" are also effective for SACP with a threshold.

**Table 16:** Empirical time complexities of ExternalAhead for  with  and selectivity.

			
0.1			
0.111			
0.125			
0.143			
0.167			
0.2			

## 5. Conclusion

In this paper, we have presented fast algorithms to find a 0-1 assignment for external nodes of a BN, which maximizes the minimum score of singleton attractors. We performed theoretical and experimental analyses for these proposed algorithms, which showed good agreements between their theoretical results and empirical results. It was also suggested that assigning internal nodes in advance of external nodes was the fastest. Furthermore, we have implemented some heuristic algorithms although theoretical analysis has not been performed. One of our future works is to extend our algorithms to a problem where it is not given which nodes are external. Furthermore, for practical use, it is important to develop a method for controlling steady states of a continuous model of biological networks. Although BN is not a continuous model, the idea based on combinatorial models may be utilized in the analysis of continuous models as in [38]. Therefore, it is also our important future work to develop a method for extending our model to continuous one.
